# Targeted deletion of the zebrafish actin-bundling protein L-plastin (*lcp1*)

**DOI:** 10.1371/journal.pone.0190353

**Published:** 2018-01-02

**Authors:** Margaret J. Kell, Rachel E. Riccio, Emily A. Baumgartner, Zachary J. Compton, Paul J. Pecorin, Taylor A. Mitchell, Jacek Topczewski, Elizabeth E. LeClair

**Affiliations:** 1 Department of Pediatrics, Northwestern University Feinberg School of Medicine / Stanley Manne Children’s Research Center, Chicago, Illinois, United States of America; 2 Department of Biological Sciences, DePaul University, Chicago, Illinois, United States of America; 3 Department of Biochemistry and Molecular Biology, Medical University of Lublin, Lublin, Poland; University of Florida, UNITED STATES

## Abstract

Regulation of the cytoskeleton is essential for cell migration in health and disease. Lymphocyte cytosolic protein 1 (*lcp1*, also called L-plastin) is a hematopoietic-specific actin-bundling protein that is highly conserved in zebrafish, mice and humans. In addition, L-plastin expression is documented as both a genetic marker and a cellular mechanism contributing to the invasiveness of tumors and transformed cell lines. Despite L-plastin’s role in both immunity and cancer, in zebrafish there are no direct studies of its function, and no mutant, knockout or reporter lines available. Using CRISPR-Cas9 genome editing, we generated null alleles of zebrafish *lcp1* and examined the phenotypes of these fish throughout the life cycle. Our editing strategy used gRNA to target the second exon of *lcp1*, producing F0 mosaic fish that were outcrossed to wild types to confirm germline transmission. F1 heterozygotes were then sequenced to identify three unique null alleles, here called ‘Charlie’, ‘Foxtrot’ and ‘Lima’. *In silico*, each allele truncates the endogenous protein to less than 5% normal size and removes both essential actin-binding domains (ABD1 and ABD2). Although none of the null lines express detectable LCP1 protein, homozygous mutant zebrafish (-/-) can develop and reproduce normally, a finding consistent with that of the L-plastin null mouse (LPL -/-). However, such mice do have a profound immune defect when challenged by lung bacteria. Interestingly, we observed reduced long-term survival of zebrafish *lcp1 -/-* homozygotes (~30% below the expected numbers) in all three of our knockout lines, with greatest mortality corresponding to the period (4–6 weeks post-fertilization) when the innate immune system is functional, but the adaptive immune system is not yet mature. This suggests that null zebrafish may have reduced capacity to combat opportunistic infections, which are more easily transmissible in the aquatic environment. Overall, our novel mutant lines establish a sound genetic model and an enhanced platform for further studies of L-plastin gene function in hematopoiesis and cancer.

## Introduction

Cell movement is essential for the immune system, but can be destructive in diseases such as cancer. How cells regulate movement is therefore an important issue. Here, we investigate a highly conserved cytoplasmic component of cell motility, the actin-bundling protein leukocyte plastin (L-plastin). This protein was originally discovered in neoplastic human fibroblasts [1, 2] and was soon identified as significantly upregulated in many cancer cell lines and solid tumors [3]. Interestingly, L-plastin is also highly expressed in normal leukocytes, including macrophages, monocytes, and neutrophils [4, review by 5]. Current research on L-plastin is thus split into two health-related fields: that of leukocyte biology, and that of cancer biology. The common theme, however, is regulation of the actin cytoskeleton and its effects on cell motility.

**Fig 1:**
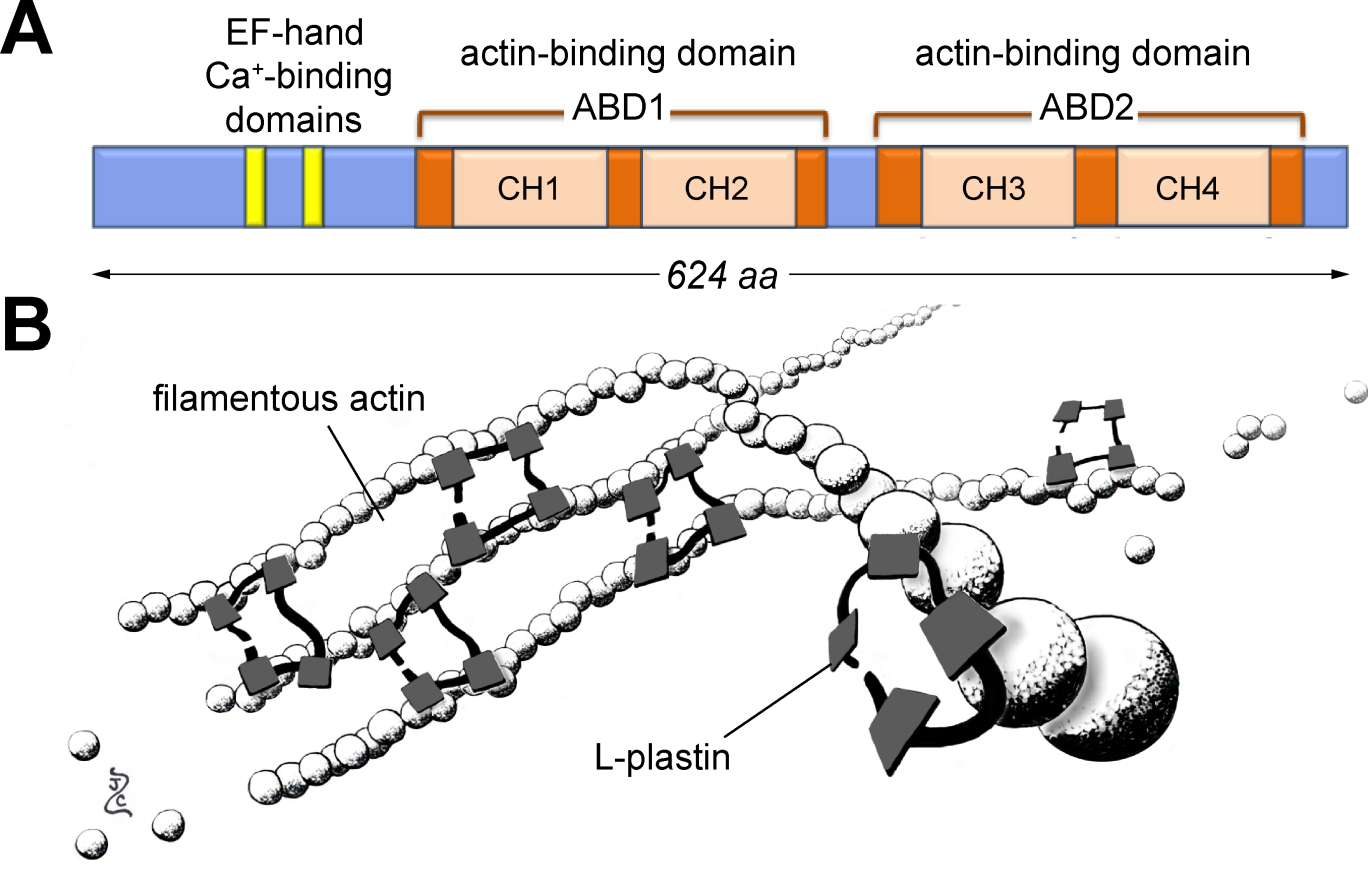
Overview of zebrafish lymphocyte cytosolic protein 1 (LCP1 or 'L-plastin'). **A)** Critical domains of the L-plastin protein. These include two EF-hand calcium-binding sites at the 5' end, and two actin-binding domains (ABD1 & 2) along the remainder of the peptide. Each actin-binding domain contains two serial calponin-homology domains (CH1 through 4). **B)** L-plastin in action. Each L-plastin monomer can bind two adjacent molecules of filamentous actin, stabilizing the parallel strands. Illustration by ZJC; L-plastin structure based on [8].

L-plastin’s short sequence has few functional domains (Fig 1A). At the N-terminus, there are 2 EF-hand calcium-binding motifs. At the C-terminus, there are two actin-binding domains (ABD1 and 2), each of which contains two calponin-homology regions (CH1 through 4). The tertiary structure of L-plastin is thought to resemble a small bundle with the ABDs on opposite sides. Each side contacts an actin filament, stabilizing the parallel strands (Fig 1B). Surprisingly, L-plastin has poor binding affinity for already-polymerized actin [6]. It is therefore proposed that the L-plastin-actin complex forms only during actin elongation, and that the sequential docking of L-plastin propagates conformational changes along the actin filaments, allowing more L-plastin to bind. This coordinated assembly may stabilize very long strands of F-actin, which are typical of highly motile or ‘probing’ cells [7].

L-plastin’s crosslinking activities regulate cell shape and behavior in several lineages of the immune system, including macrophages, neutrophils, B-cells and T-cells [9–13] [review by 14]. Conversely, aberrant L-plastin expression is a hallmark of cancer [15]. Experimental activation of L-plastin can enhance the proliferation, invasiveness and lethality of tumor cells both *in vivo* and *in vitro*, whereas suppression can reverse these characteristics [16–22]. Because of its limited expression in normal cells and mechanistic role in many malignancies, L-plastin has been frequently studied as a cancer diagnostic marker [23], prognostic marker [24], or therapeutic target [25, 26]. However, much about L-plastin gene regulation and protein function is still unknown.

Experiments disabling specific genes in zebrafish have been essential for defining the role of DNA mutations in development and disease. Although antisense RNAs or morpholino oligos can interrupt gene function for a short time, germline transmission of knockout alleles is the most stable method for examining phenotypes throughout the life cycle. Because there are no knockouts, mutants, or transgenics for zebrafish L-plastin, we set out to produce a targeted modification of this locus, making a new animal model for the study of plastin function. Gene editing in zebrafish is now highly efficient, allowing the rapid establishment of mutant lines [27, 28]. Zebrafish leukocytes develop within 48 hours of fertilization [29] and there are several transgenics that fluorescently label specific cell lineages, such as macrophages and neutrophils [30]. Finally, the zebrafish is a popular biological model for cancer studies, allowing single-cell analysis of how transformed cells interact with host tissues [31, 32]. All of these features make the zebrafish an effective platform for the study of cell motility *in vivo*, and an attractive context for testing the cell-motility consequences of plastin mutations.

In this report we provide an updated phylogenetic analysis of the zebrafish plastin genes (L-, T- and I-plastin), in comparison to their mammalian orthologs. Although many genes in fishes appear as duplicate paralogs, all of the zebrafish plastin proteins are shown to be single-copy sequences, facilitating targeted deletion and direct comparison with mammalian models. Using CRISPR/Cas9 reagents designed against zebrafish L-plastin (*lcp1*), we established three independent null alleles here called ‘Charlie’ (CH), ‘Foxtrot’ (FX) and ‘Lima’ (LM). Incrossed heterozygous fish (*lcp1* +/-) produced viable embryos of all expected genotypes; however, by both whole-mount immunohistochemistry and Western blotting, LCP1 protein was undetectable in null embryos and adults. We saw no gross morphological defects in either maternal or maternal-zygotic mutants, and both heterozygous and null organisms were able to develop and reproduce normally. This phenotype is consistent with that of the L-plastin null mouse [LPL -/- 33, 34]; however, such mice do have a profound immune defect when challenged by bacteria. Interestingly, we observed reduced long-term survival of zebrafish *lcp1 -/-* homozygotes in all three of our independent CRISPR knockout lines, amounting to ~30% below the population expectation. Given the known immunodeficiency of the null mouse, this suggests that the null zebrafish may also be susceptible to opportunistic infections, which are more easily transmissible in the aquatic environment. Overall, our novel mutant lines establish an enhanced model for further insights into L-plastin gene function in vertebrate leukocyte development, immune function, and cancer.

## Results

### Zebrafish have single protein orthologs of all three mammalian plastin proteins

The evolutionary relationships of the plastins are difficult to discern for both biological and organizational reasons. Biologically, the vertebrate plastins have radiated into three distinct sequences (I-plastin, L-plastin, and T-plastin), but these retain high nucleotide and amino acid similarities, particularly in the actin-binding domains. Organizationally, the nomenclature of the plastins is confused by multiple mismatched sets of alphanumeric identifiers. In nucleotide databases the human L-plastin gene is called LCP1; however, in protein databases it is called ‘plastin 2’. Such mismatches hinder accurate identification of plastin sequences across species, impeding the study of these highly conserved proteins.

To generate an updated evolutionary tree for the plastin protein family, we began with the most recent zebrafish genome assembly (Ensembl GRCz10). We selected the complete mRNA for our zebrafish target gene, lymphocyte cytosolic protein 1 (*lcp1*; NCBI RefSeq NM_131320.2) and used it as a nucleotide-to-protein query using BLASTx [35, 36]. Protein matches were limited using the “model organisms” option, plus an arbitrary cutoff of >50% amino acid similarity to the zebrafish query. Of all the results, only the top 25 matches were analyzed. After discarding all “predicted” protein entries and several within-species isoforms, 9 sequences representing selected vertebrates (*D*. *rerio*, *M*. *musculus*, and *H*. *sapiens)* and 6 sequences representing other metazoans (*C*. *elegans*, *D*. *discoideum*, and *D*. *melanogaster*) were used to generate a maximum-likelihood phylogenetic tree. A detailed phylogenetic workflow is provided in the Methods.

**Fig 2:**
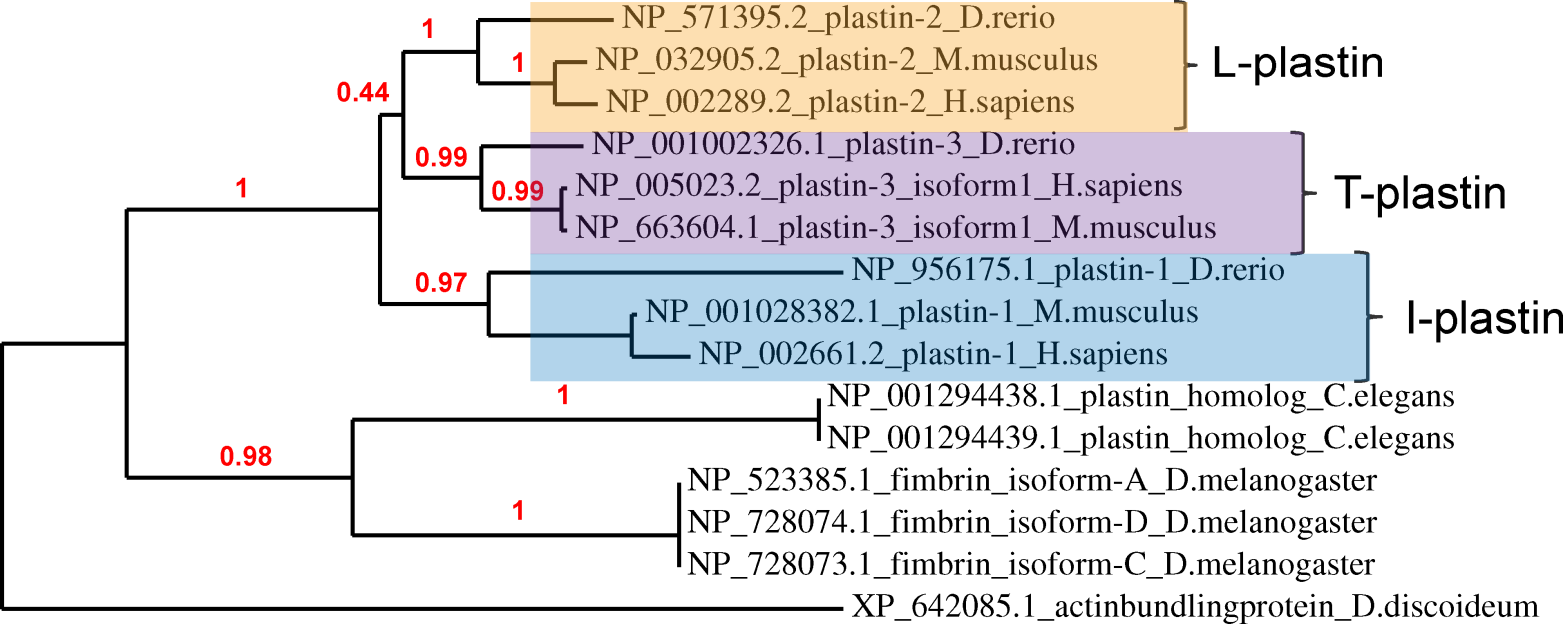
Zebrafish orthologs of the plastins. Maximum-likelihood phylogenetic analysis of plastin proteins from selected metazoans. All proteins shown were retrieved by mRNA-to-protein search (BLASTx) using the zebrafish L-plastin mRNA (ZDB-GENE-991213-5, NCBI RefSeq NM_131320.2) as a query. A calculated confidence level (percent of 100 bootstrap replicates) supports each node. For details of phylogenetic reconstruction, see Methods.

Our completed tree (Fig 2) shows that vertebrate plastin proteins form a crown group in which there are three major isoforms. Using the current protein nomenclature, these are called plastins 1, 2 and 3. There was strong branch support for each plastin as a separate family (> 97% of bootstrap replicates), but less support for the sister-group relationship of plastin 2 to plastin 3 (< 50%). Notably, there was only one zebrafish protein per isoform, which was always placed as the outgroup to the mouse and human versions.

**Fig 3:**
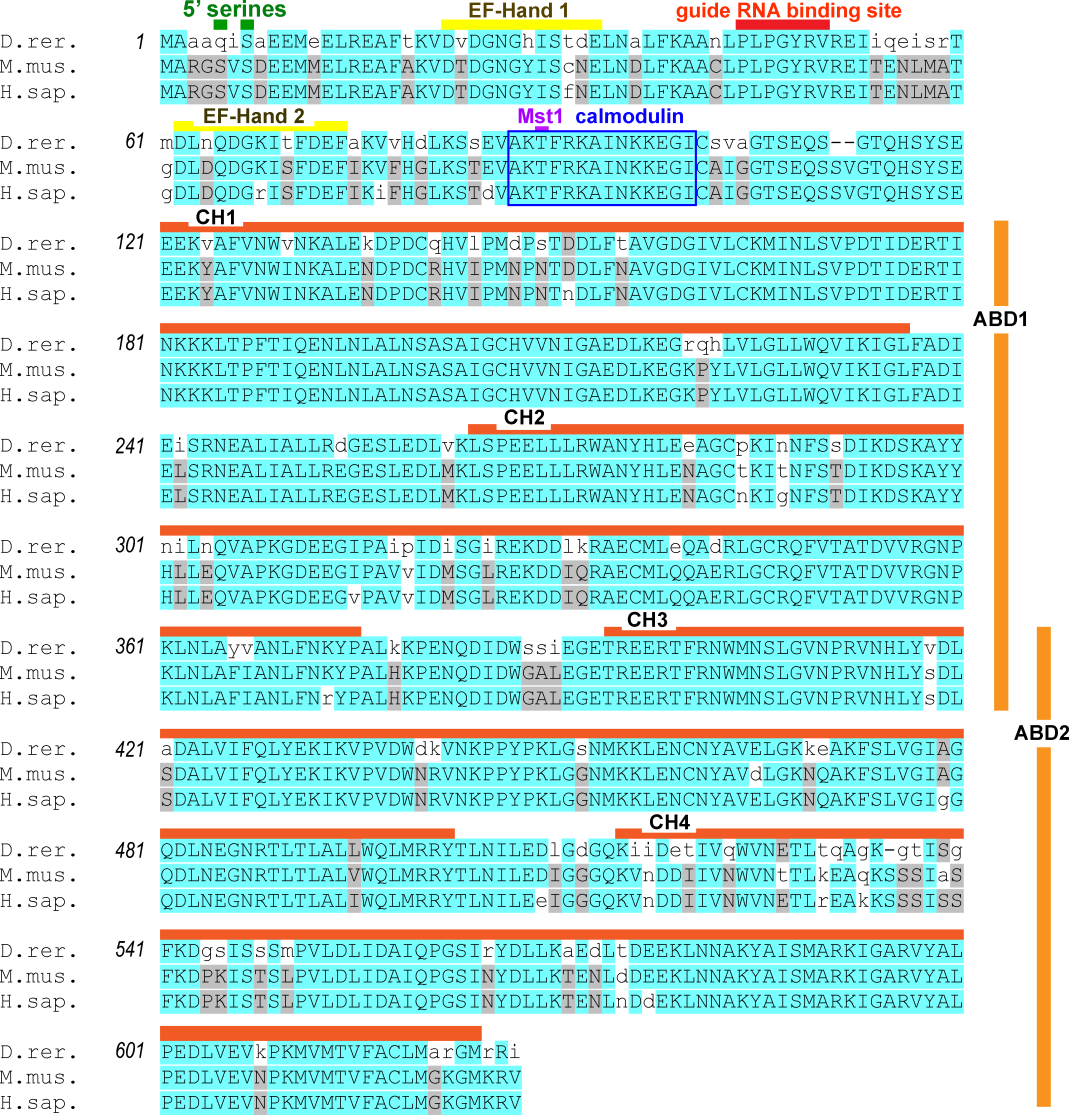
Amino acid alignment of the zebrafish, mouse and human L-plastin (‘plastin 2’) proteins. Zebrafish, mouse and human plastin 2 proteins were aligned using MUSCLE. *D*. *rer*. = NP_571395, *M*. *mus*. = NP_032905, and *H*. *sap*. = NP_002289. Colored bars indicate key protein residues or motifs. Amino acids are shaded according to alignment scores derived from the default blocks amino acids substitution matrix (BLOSUM). High BLOSUM scores (blue) reflect alignments that closely match the consensus and are highly improbable in a random model. ABD = actin-binding domain; CH = calponin-homology domain; Mst = mammalian Ste20-related protein kinase site.

Examining our query sequence, the closest translational match to the zebrafish *lcp1* mRNA was the zebrafish protein NP_571395.2, called ‘plastin 2’. The mammalian orthologs of this protein are the mouse and human plastin 2 proteins, encoded by the genes lcp1 and LCP1, respectively. Fig 3 shows the alignment of these three proteins. Overall, zebrafish L-plastin is 83% and 82% identical to the mouse and human sequences, respectively. The zebrafish protein is three amino acids shorter than the mammalian versions (624 vs. 627 residues), spread between two gaps. One gap, which deletes a serine-valine (SV), is at the 5’ end of the protein and outside any known functional domain. The other gap, a single serine (S), is at the 3’ end of the protein in calponin-homology domain 4.

The zebrafish sequence shares with mammals several post-translational modification sites known to be important in L-plastin function. In the 5’ headpiece of the protein, the zebrafish differs in the substitution of a glutamine for a serine at position 5 (Ser5Glu), whereas the mouse and human share two serines (Ser5 and Ser7). In mammals, the phosphorylation of Ser5, Ser7 or both is known to increase L-plastin localization to the cytoskeleton, filament-bundling activity and the invasiveness of both melanoma and breast cancer cell lines [20, 21, 37, 38]. Zebrafish, mouse and human L-plastin do share a threonine at position 89 (T89), a confirmed site of Mst1 phosphorylation [39, 40]. Finally, all three sequences have an identical 14-amino acid calmodulin- binding domain (AKTFRKAINKKEGI), suggesting conserved regulation by this calcium-binding protein [41].

Overall, our *in silico* analysis indicated that zebrafish have a single protein sequence corresponding to each of the three plastins found in humans and mice (Table 1). Of these, L-plastin appeared to us to be an attractive target for gene knockout. We therefore planned a CRISPR/Cas9 gene-editing strategy to modify this locus.

**Table 1:** The plastin gene family in zebrafish, mice and humans.

Species	Protein Identifier	Protein Name	mRNA identifier	Gene Name	Chromosome #
*D*. *rerio*	NP_571395.2	plastin 2	NM_131320.2	lcp1	9
	NP_956175.1	plastin 1	NM_199881.1	pls1	2
	NP_001002326.1	plastin 3	NM_001002326.1	pls3	14
*H*. *sapiens*	NP_002289.2	plastin 2	NM_002298.4	LCP1	13
	NP_002661.2	plastin 1	NM_002670.2	PLS1	3
	NP_005023.2	plastin 3	NM_005032.6	PLS3	X
*M*. *musculus*	NP_032905.2	plastin 2	NM_008879.4	Lcp1	14
	NP_001028382.1	plastin 1	NM_001033210.3	Pls1	9
	NP_663604.1	plastin 3	NM_145629.2	Pls3	X

### CRISPR/Cas9 targeting of zebrafish L-plastin (lcp1)

**Fig 4:**
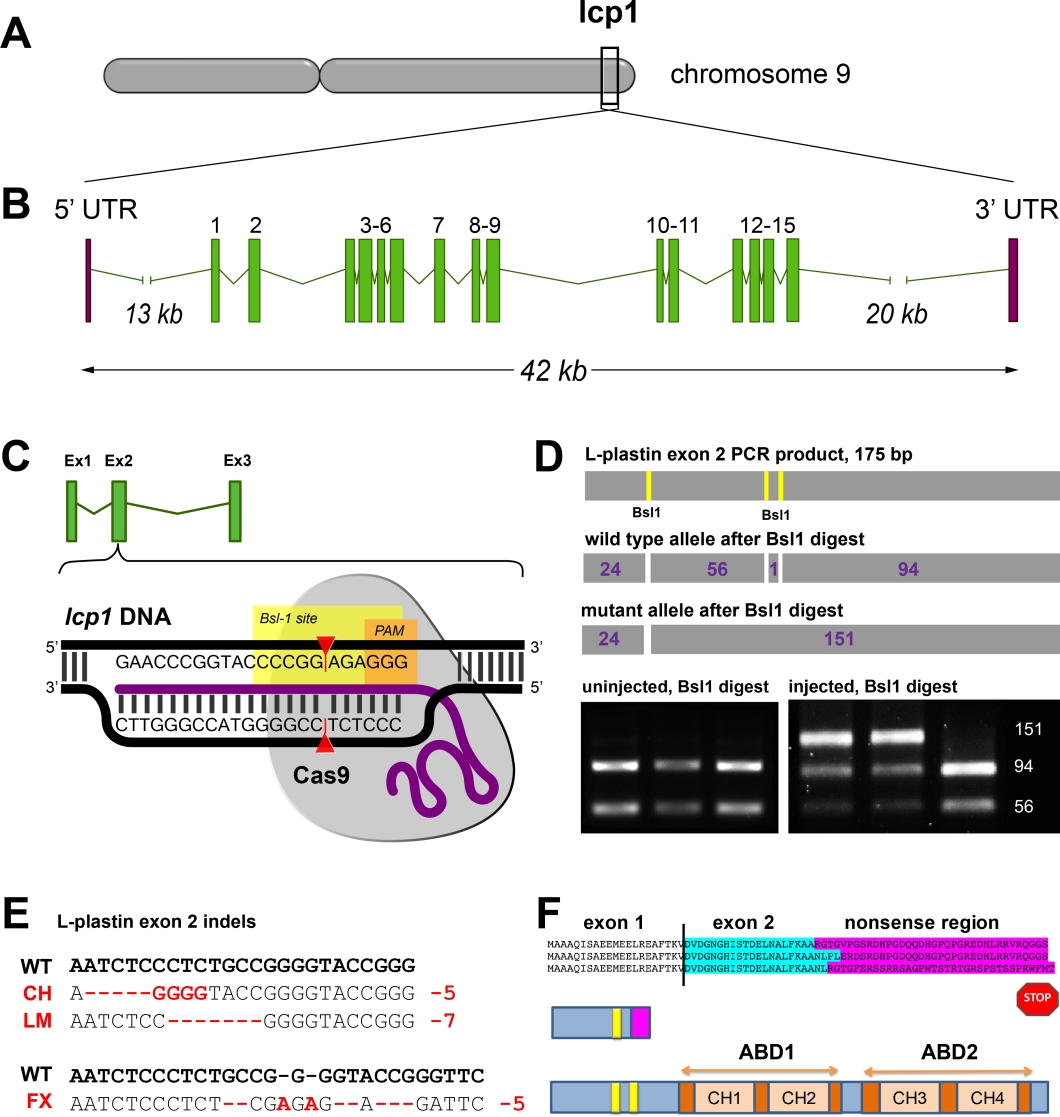
CRISPR/Cas-9 targeting of zebrafish lcp1 exon 2. **A)** Location of the L-plastin locus on zebrafish chromosome 9. **B)** Exon-intron structure of the locus (~42 kB). The most 5' and 3' exons are untranslated (UTR) and are separated from the intervening coding exons (here numbered 1 through 15) by large introns. **C)** Schematic of the CRISPR/Cas9 target site within zebrafish *lcp1* exon 2. The exon 2 guide RNA (purple) binds the complementary genomic DNA (black) and docks with the Cas9 nuclease (gray). Upstream of the required protospacer-adjacent motif (PAM, in orange), the Cas9 cleavage site targets a *Bsl*1 restriction site (yellow), causing indels. **D)** PCR genotyping of gene-edited *lcp1* exon 2. Top row: PCR primers flanking the PAM amplify a 175-bp product with three *Bsl*1 sites. Second row: An unedited PCR product digested with *Bsl*1 produces four fragments. Third row: An edited PCR product produces three fragments, with the large (156 bp) band being diagnostic. Bottom left: PCR genotyping of three uninjected embryos (24 hpf), showing a wild-type pattern. Right panel: PCR genotyping of three injected embryos, showing evidence of gene editing in the two leftmost lanes. **E)** Alignment of wild type and edited alleles of zebrafish *lcp1*. CH = ‘Charlie’, a 5 bp deletion; LM = ‘Lima”, a 7-bp deletion; FX = ‘Foxtrot’, a net 5 bp deletion. **F)** Predicted frame shifts, stop codons and truncated peptides derived from the mutant alleles. All predicted proteins are < 5% of the full-length version (image not to scale), and lack both actin-binding domains (ABDs).

Our first guide RNA (gRNA) was designed against *lcp1* exon 2, which is the larger of the first two coding exons (Fig 4A and 4B). This exon provided adequate sequence to design a gene-specific guide RNA, but was positioned well upstream of the exons encoding the actin-binding domains (ABD1 and 2). Within exon 2, we identified a *Bsl*1 restriction site just upstream of a protospacer-adjacent motif, or PAM. We therefore chose this particular motif for our experiments. Fig 4C depicts the intended interaction of genomic DNA, guide RNA and Cas9 protein in this region.

24 hours after injection of a standard CRISPR/Cas9 reaction mix into wild type zygotes, we extracted genomic DNA from early embryos. To assess gene-editing efficiency, we used PCR amplification of the exon 2 target region, followed by a *Bsl*1 restriction assay (Fig 4D). Table 2 provides the primer sequences used in this assay, and Table 3 the expected restriction fragments. All uninjected embryos showed the baseline restriction pattern, which appears as a doublet. However, in most of the injection experiments over half of the embryos showed a triplet restriction pattern, indicating successful genome editing. From this we inferred that our guide RNA had moderate double-strand break activity against the intended region of *lcp1* exon 2, and we used the same dose of this gRNA for all subsequent experiments.

**Table 2:** Key oligos and primers.

Primer Name	Primer Sequence
*lcp1* exon 2 guide RNA forward	5'- ATT TAG GTG ACA CTA TAG AAC CCG GTA CCC CGG CAG AGT TTT AGA GCT AGA AAT AGC -3'
*lcp1* exon 2 genotyping forward	5'- gaa ggt gat ctt ccc gtc ct -3'
*lcp1* exon 2 genotyping reverse	5'- gcc tga cct ttg acc ttg tc -3'

**Table 3:** Diagnostic restriction enzyme digests and fragments produced.

	*Bsl*1 digest*	*Mnl*1 digest*
	Charlie (CH), Foxtrot (FX)	Lima (LM)
wild type (+/+)	94, 56, 24, 1	102, 56, 17 bp
heterozygote (+/-)	145, 94, 56, 24, 1	151, 102, 56, 17 bp
null (-/-)	145, 24, 1	151, 17 bp

* The underlined bands are most easily visible and thus diagnostic. Smaller fragments may be present, but are harder to see.

By repeating these procedures for several months, we established 21 “generation zero” (G0) mutagenized fish lines, each comprising surviving siblings all injected at the 1–2 cell stage. When these injected fish matured, we screened 8–10 fish per line by fin-clips for evidence of mosaicism; only strong mosaics were retained. We then outcrossed confirmed G0 mosaics with wild types and genotyped 8–16 embryos per F1 clutch at 1–2 days post fertilization. The G0 parent was classified as a germline transmitter if some proportion of its offspring had a triplet restriction pattern after genotyping PCR. Out of 252 F1 embryos screened by this method, only 41 (16%) inherited an exon 2 mutation. Despite repeated crossing attempts, 11 of the G0 lines did not transmit any modified alleles (52% of lines). Within the 10 lines that did transmit (48% of lines), the proportion of mutated offspring per clutch varied considerably, from 5–75% (median = 29%). These results suggest broad germline heterogeneity among the G0s, as expected when generating mosaic animals. Nevertheless, we saw sufficient penetrance of heritable mutations to screen ten independent lines for possible knockout alleles.

To propagate the F1s, we raised surviving embryos to sexual maturity, then genotyped by fin-clip to isolate heterozygotes. In the final round, selected *lcp1* +/–fish were outcrossed to wild types, producing a third generation (F2). All F2 clutches showed 50% inheritance of a modified *lcp1* allele (not shown), confirming Mendelian inheritance *in vivo*.

### Cloning and sequencing of the L-plastin mutant alleles

After CRISPR/Cas9 double-strand breaks, DNA is most commonly repaired by non-homologous end-joining [NHEJ 42, 43]. Therefore each application of CRISPR/Cas9 has the potential to produce unique local indels, and thus different alleles. To identify the *lcp1* alleles produced by our injections, we subcloned and sequenced the exon 2 PCR products from confirmed F1 heterozygotes in 3 of the 10 germline-transmission lines. Each of these 3 lines yielded a unique mutation, which we named according to the “call sign” of the parent line: Charlie, Foxtrot, and Lima. Charlie (CH) is a 5 bp deletion / 4-bp substitution. Foxtrot (FX) is complex indel/substitution series resulting in a net 5-bp deletion. Finally, Lima (LM) is a simple 7 bp deletion. Fig 4E shows the base-pair alignments of mutant and wild type alleles. It was discovered when genotyping the LM line that the *Bsl*1 restriction digest was not reliable due to an NHEJ-induced change in the restriction site pattern. We therefore developed an alternative digest for this line using a different enzyme, *Mnl*1 (Table 3). However, the general diagnostic patterns of wild type doublet and mutant triplet were maintained, allowing efficient screening of individual genotypes by PCR.

Although different in size and sequence, all 3 of our *lcp1* indels are predicted to cause severe premature truncation of the zebrafish L-plastin protein. Downstream of the PAM in exon 2, translation shifts to a short sequence of 30–35 missense amino acids, followed by a stop (Fig 4F). Using the predicted missense peptides as BLASTp queries against all known proteins, we detected no matching proteins or motifs that would suggest off-target functions. Thus, all of the sequence-confirmed CRISPR lines are predicted to produce short, non-functional LCP1 peptides representing less than 5% of the normal protein and lacking both actin-binding domains. We therefore predicted that all three gene-edited lineages (CH, FX and LM) might produce equivalent L-plastin null phenotypes, and our subsequent analyses sought to test this hypothesis in one or more lines.

### Confirmation of L-plastin knockout in embryos and adults

We next assessed whether L-plastin protein could be detected in null animals (*lcp1* -/-). The zebrafish immune system develops rapidly, and post-hatching embryos have abundant macrophages and neutrophils [44, 45]. L-plastin is highly expressed in the cytoplasm of such cells, and so the absence of L-plastin in these lineages would be consistent with a successful gene knockout.

**Fig 5:**
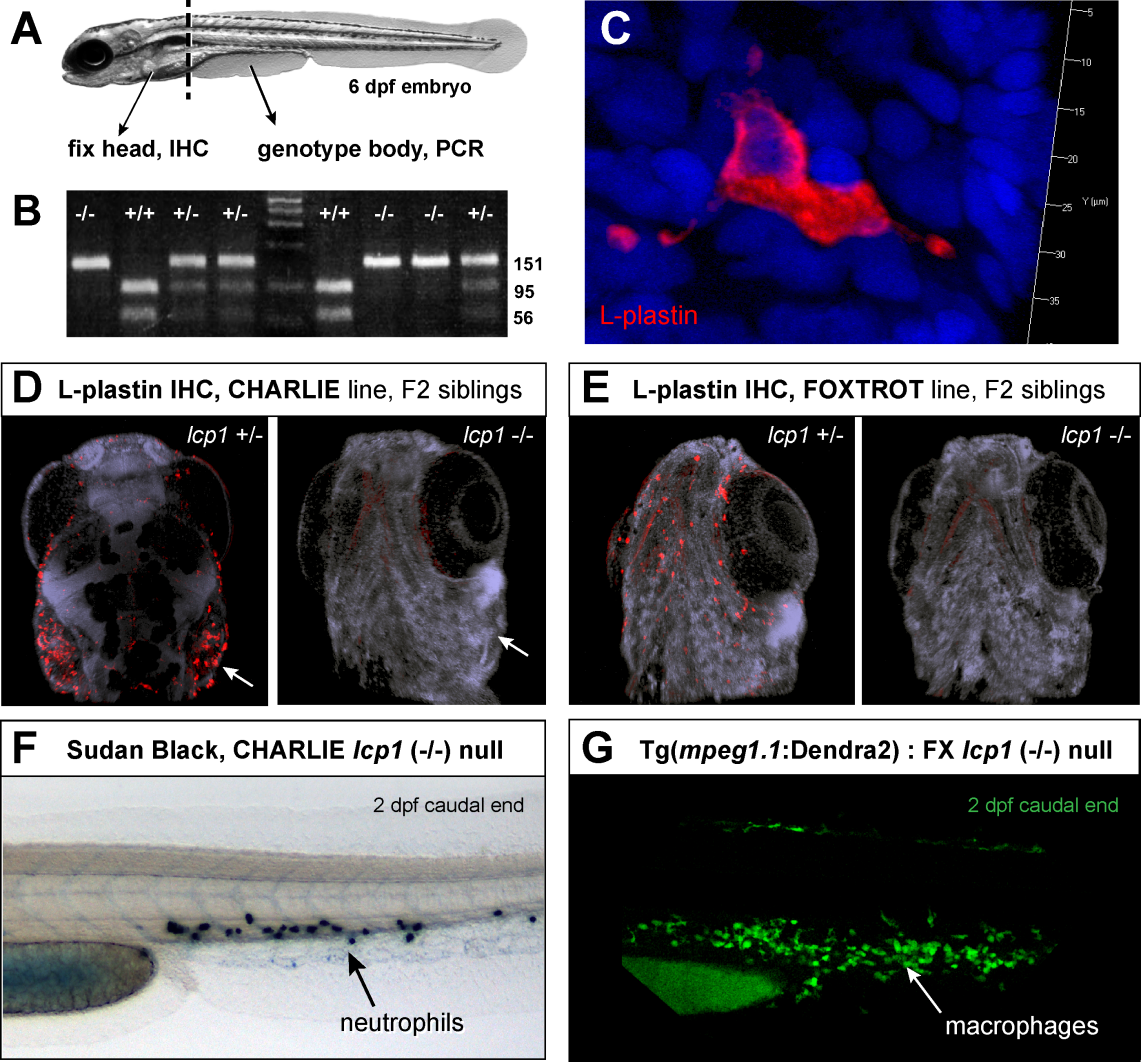
Leukocyte staining is abolished in *lcp1* null animals. **A)** Flow chart of the experiment. *lcp1* incrosses (+/- x +/-) were raised to 6 dpf and then processed for whole-mount immunostaining (head) and genomic DNA isolation (body). **B)** Representative genotyping results after genotyping PCR and *Bsl*1restriction enzyme digest. Homozygous wildtype, homozygous null, and heterozygous siblings are easily distinguished. **C)** Typical fluorescent immunostaining of a superficial leukocyte in wildtype and heterozygous animals. There is intense signal in the entire cytoplasm, including distant cellular projections; in contrast, the nucleoplasm is dark. **D)** Heterozygous and null siblings of the Charlie line (CH). In heterozygous fish, leukocytes are stained intensely, particularly in the gill area (arrow). In null fish, no leukocytes are visible. A faint, non-specific staining is present in all embryonic skeletal muscle. **E)** Heterozygous and null siblings of the Foxtrot line (FX). In the null animal, the LCP1-positive cells are undetectable. **F)** Null animals have neutrophils as seen in 2 dpf caudal fins stained with Sudan Black. **G)** Null animals have macrophages, as seen in 2 dpf caudal fins from embryos with green macrophages (Tg(*mpeg1*.*1*:Dendra2)). Nuclei are counterstained with DAPI.

We established incrosses from *lcp1* heterozygous parents (*lcp1* +/- x *lcp1* +/-) and raised multiple clutches to 5–6 days post-fertilization. 16–20 embryos per clutch were collected, and each fish was then bisected to fix the head and genotype the trunk (Fig 5A). After PCR, we detected all expected genotypes in the trunk samples (*lcp1* +/+, +/-, and -/-; Fig 5B), allowing us to sort the heads into three groups for antibody staining. For our primary antibody, we used an established rabbit polyclonal designed against the first 111 amino acids of zebrafish L-plastin [46–49, see experiments in 50]. This reagent allowed us to detect hundreds of strongly-stained amoeboid cells in the superficial tissues of both wild type and heterozygous animals (Fig 5C). The pattern and intensity of staining was similar in these two groups (not shown), leading us to compare the staining patterns between heterozygotes and nulls. In this case, the intense, punctate staining seen in heterozygotes was absent in all homozygous mutant siblings, consistent with the predicted truncation of L-plastin protein (Fig 5D and 5E). A faint stain was detected in the larval striated musculature, which contains abundant actin. This signal did not differ between genotypes, so was considered non-specific in this assay.

Because L-plastin expression is restricted to leukocytes, deleting this protein removes one of the most commonly-used markers of immune cells. To confirm that the loss of L-plastin immunoreactivity was not due to leukocyte depletion, we used alternative methods to detect both neutrophils and macrophages in our mutant lines. By applying the histochemical substrate Sudan Black, which reacts with the myeloperoxidase in neutrophil granules, we detected in 2 dpf null embryos abundant neutrophils in the caudal hematopoietic tissue (Fig 5F). Likewise, after independently crossing two of our L-plastin null alleles (CH and FX) for two consecutive generations into a transgenic background expressing a macrophage-specific green fluorophore (Tg(*mpeg1*.*1*:Dendra2)), we saw in compound null / transgenic animals numerous labeled cells in the expected areas (Fig 5G). We conclude that the loss of L-plastin staining in null animals is due to reduced or absent target protein, and not the complete absence of early immune cells. Therefore *lcp1* -/- mutant zebrafish should be a valid model in which to study the consequences of L-plastin deficiency in macrophages, neutrophils, and other hematopoietic cells types that normally express this peptide.

**Fig 6:**
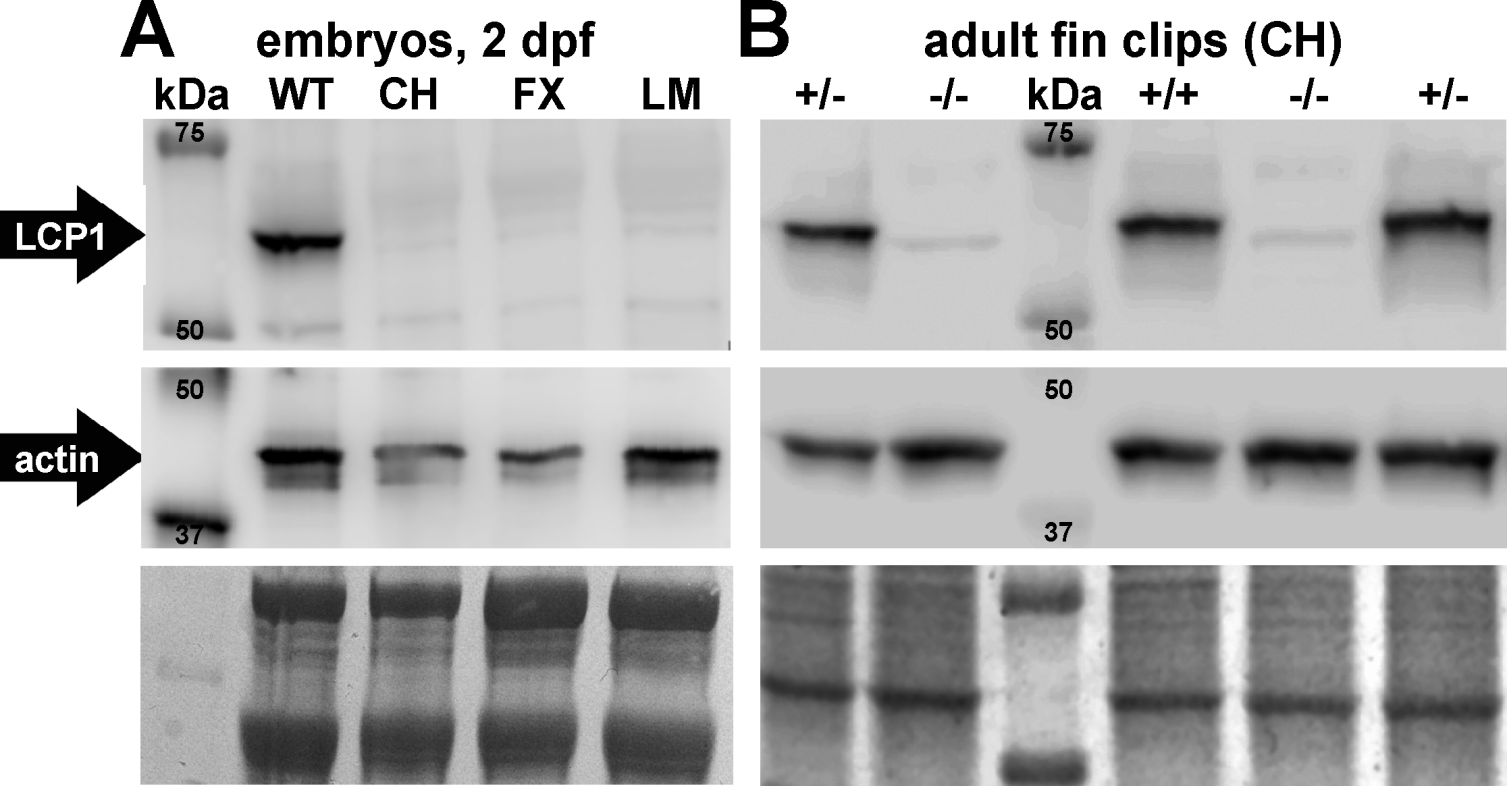
LCP1 protein is undetectable in null embryos and adults. **A)** Chemiluminescent Western blot comparing 2 dpf total protein lysates from a wild type line (WT) and three independent null lines (CH, FX and LM). The top panel shows LCP1 signal (~65 kDa) and the middle panel shows actin signal (~ 43 kDa). The bottom panel reflects the total protein in each lane (Coomassie Blue). **B)** Chemiluminescent Western blot comparing 5 adult animals of one line (CH): two heterozygotes (+/-), one wild type (+/+), and two nulls (-/-).

To increase the sensitivity of our protein detection, we next made Western blots of total protein lysates from 2 dpf wild types and 2 dpf homozygous mutant embryos (*lcp1* -/-) from each of our three mutant lines (CH, FX and LM). Consistent with our embryonic immunostaining, the ~65 kDa L-plastin band was expressed strongly in wild type controls, but was undetectable in maternal-zygotic nulls (Fig 6A). All embryo lysates were positive for actin (~ 43 kDa). Approximately equal loading and transfer conditions were confirmed by post-detection staining with Coomassie Blue.

We next wanted to examine if L-plastin protein was also absent from null zebrafish at later stages of development. To do this, we repeated Western blotting on total protein lysates of caudal fin tissue from five genotyped adult fish of the Charlie line. All three *lcp1* genotypes were represented (one CH +/+, two CH +/-, and two CH -/-) and the sample lanes were loaded in a random order on the gel. Lysates from wild types and heterozygotes were both positive for L-plastin, and the intensity of this band was similar in both groups. This suggests that our engineered mutations are likely recessive, and can be compensated by one normal allele. In lysates from null fish, no specific detection was observed (Fig 6B). Finally, semi-quantitative reverse-transcription polymerase chain reaction (RT-PCR) experiments confirmed significantly lower detection of *lcp1* transcripts in null animals relative to wild types (not shown), a result consistent with known mechanisms of nonsense-mediated mRNA degradation.

Taken together, these experiments strongly suggest that all three of the *lcp1* mutant alleles predicted to cause premature truncation *in silico* abolish protein expression *in vivo*, and that individual zebrafish homozygous for such alleles lack L-plastin protein throughout the life cycle.

### Long-term survival of lcp-1 knockout zebrafish is reduced

At this point, we knew that L-plastin null zebrafish could be viable and fertile. However, we also became aware of a mouse knockout of L-plastin, in which there are both subtle and severe immune deficiencies. Mice lacking L-plastin (LPL -/-) have impaired T-cell and B-cell activation, as well as decreased production of T-cell-dependent antibodies [11, 51]. However, the most dramatic defect is the inability of lung macrophages to clear certain infections. When exposed to sub-lethal doses of pulmonary pneumococcal organisms, null mouse pups die rapidly due to defective migration, maturation and retention of alveolar macrophages [9, 52]. We therefore reasoned that *lcp1* loss-of-function in zebrafish could compromise resistance to infection, particularly in the post-hatching stages when fry are free-swimming, but before their adaptive immune systems are fully mature. In zebrafish, it requires 3 weeks for differentiated T-cells to leave the thymus, and 4–6 weeks for T- and B-cells to produce effective humoral responses to antigens [53]. Thus, we designed an experiment to test if survival beyond 6 weeks post-fertilization was affected in zebrafish lacking LCP1, a known component of leukocyte motility.

**Fig 7:**
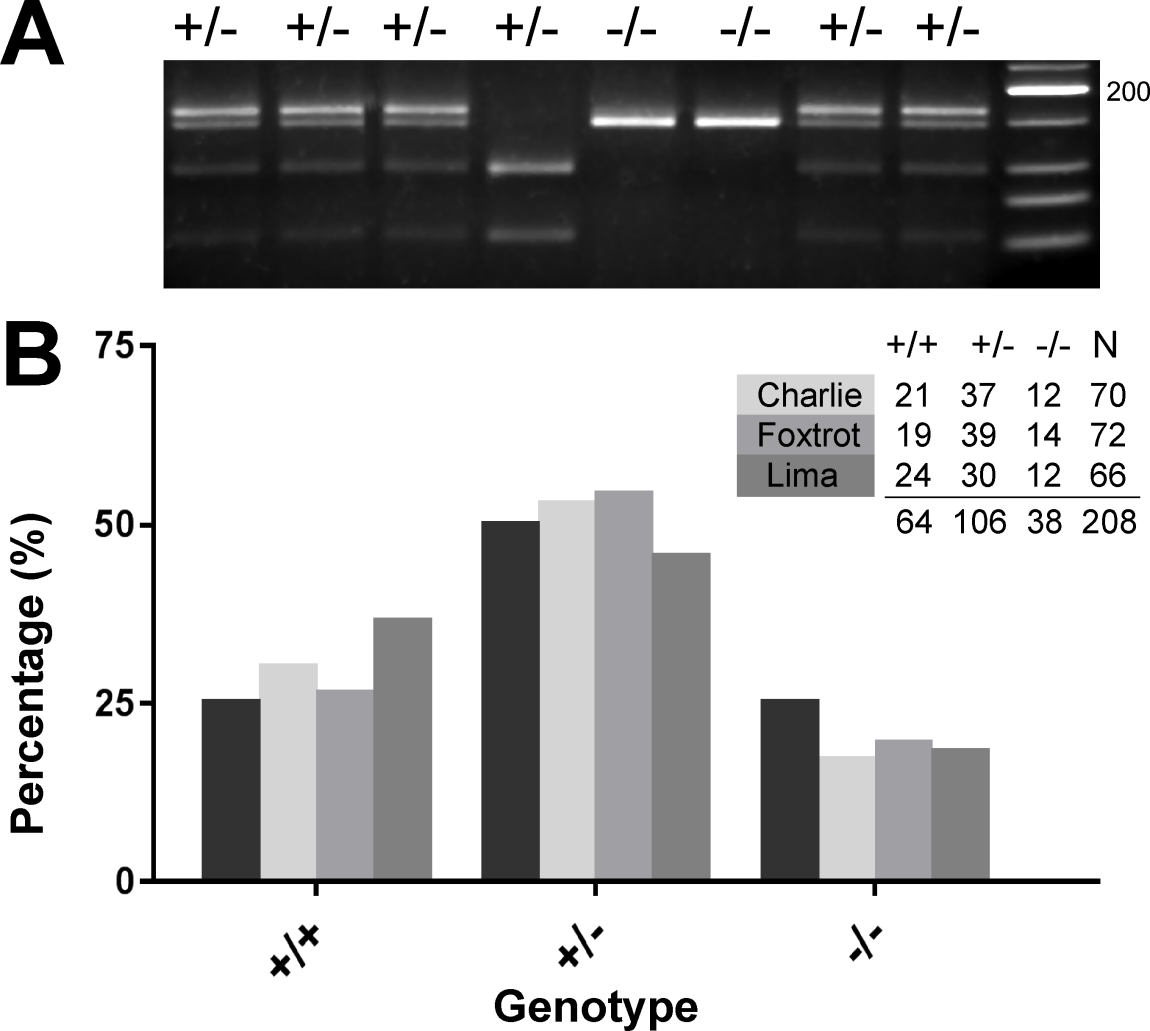
Survivorship analysis of lcp1 allele carriers. **A)** Lima (LM) genotyping gel showing representative *Mnl*1 digest products against a low molecular-weight DNA ladder. The expected banding patterns for heterozygotes (+/-), wild types (+/+) and nulls (-/-) were all observed (see Table 3). **B)** Genotype distributions for the Charlie (CH), Foxtrot (FX), and Lima (LM) lines. All surviving fish were genotyped at either 7–8 weeks (CH and FX) or >1 year of age (LM). The black columns in each cluster represent the percentages expected for each genotype (25%, 50%, or 25%); the lighter columns represent the percentages observed. Counts and sample sizes are provided in the inset table.

To test the long-term survival of *lcp1* mutant fish in our facility, we set up independent incrosses for each line (*lcp1* +/- x *lcp1* +/- for CH, FX and LM) and grew all of the surviving progeny. Two of the crosses (CH and FX) were collected after 7–8 weeks, while the third (LM) was collected after 1 year. At the time of collection, each surviving fish was individually genotyped (Fig 7A), and the observed genotype frequencies were compared to the expected Mendelian frequencies using a chi-square goodness-of-fit (Fig 7B). In all three lines there was a consistent loss of nulls, meaning that the counts of these animals were 20 to 30% below expectation (CH -/-, -31%; FX -/-, -22%; LM -/-, -27%). Notably, the deficits in the line raised for one year were not radically different from the lines raised for 7–8 weeks, leading us to infer that most of the apparent mortality occurred prior to the earlier collection. When analyzed by individual line, the deviations were not statistically significant (N_CH_ = 70; χ^2^ = 2.54, p = 0.280; N_FX_ = 72; χ^2^ = 1.19, p = 0.550; N_LM_ = 66; χ^2^ = 4.91, df = 2, p = 0.086). However, when all three lines were combined, a slight effect emerged (N_total_ = 208, χ^2^ = 6.58, p = 0.037). Against the expectation of 25% null survivorship, we observed a final proportion of only 18% (38 / 208; 95% confidence interval 13.6–24.0%). This long-term survey of L-plastin allele carriers indicates that null animals can mature in large numbers, but may have reduced survival within the first 6–7 weeks, when reared together with their wild type and heterozygous siblings.

## Discussion

### Model metazoans for cytoskeletal studies

Many cellular processes depend on the actin cytoskeleton, including division, secretion, and directed migration. Regulation of actin is thus essential for cell function. Such regulation is accomplished by a swarm of actin-modifying proteins, including assemblers, disassemblers, linkers, and bundlers. Within the actin-bundling proteins, the fimbrin/plastin family is highly conserved in metazoans, with representatives in *Arabidopsis* [54, 55], *Saccharomyces* [56] and *C*. *elegans* [57]. Here, with the production of the first L-plastin mutant zebrafish, we introduce a second vertebrate model for the study of these proteins. This novel biological resource complements the existing null mouse line [LPL -/-, 33] and provides additional opportunities to investigate cell migration in a rapidly developing and optically transparent organism.

### Conservation and divergence of fish and mammalian plastins

Most teleost fishes–including medaka, stickleback, and zebrafish–have in their evolutionary history a whole-genome duplication that causes most ancestral vertebrate genes to be represented as paired paralogs [58, 59]. These paralogs, being quite alike in sequence and product, can complicate both knockdown and knockout methods in fish biology. With respect to the plastins, however, zebrafish and mammals appear to share an ancestral, three-member family. This conservation allowed us to effectively target zebrafish L-plastin using only one guide RNA against a single locus.

Considering the alignment of the zebrafish, mouse and human L-plastin proteins, amino acid identity is quite high (>85%), suggesting that protein structure and function are likely conserved. Known regulatory motifs within human and mouse L-plastin include two phosphorylatable serines (S5 and S7), [20, 21, 37, 38], a Mst phosphorylation site [39, 40] and a calmodulin-binding domain [41]. The zebrafish protein sequence is identical in all of these features except for the position 5, which carries a glutamine (Q5; Fig 3) instead of a serine. Both serine and glutamine carry uncharged, polar side chains, though the glutamine’s is much larger. However, as L-plastin has not yet been studied in zebrafish beyond its use as a leukocyte marker, the effect of this amino acid substitution remains to be explored. Another important post-translational modification of L-plastin is its response to calcium, which activates a cryptic ‘switch helix’ between the EF-hand and ABD domains [60]. This recently-described protein folding phenomenon can now be studied in both the mouse and zebrafish models.

There are many actin cross-linkers, but most are ubiquitous. How L-plastin is restricted to specific cell types, such as leukocytes or malignant cells, is still unclear. Progress on this issue will likely come from comparative analysis of the vertebrate genomes available. Promoter features found in mammals include several steroid-hormone and transcription-factor binding sites, which may regulate L-plastin expression [61, 62]. To date there are no studies of the zebrafish promoter, but conserved similarities may yet yield new information on important regulatory motifs and domains. This regulation would have important implications for directed gene expression to leukocytes and/or tumor cells.

### Wet vs. dry: Development and viability of fish and mice lacking L-plastin

Although there are no zebrafish paralogs of *lcp1* to provide redundant functions, zebrafish embryos lacking both maternal and zygotic L-plastin can develop normally. Null fish have no gross morphological defects, and, in the laboratory, they survive and breed well. This phenotype is similar to that described for the L-plastin null mouse (LPL -/-). Such mice are indistinguishable from wild type littermates and, under normal conditions, “approximately 25%” survive" [51]. The similarity ends, however, when null pups are challenged with pathogenic lung bacteria [9]; under these conditions, 80% of wild types survive, but 80% of nulls die. The interesting mechanism of this susceptibility has been described only recently, and involves poor transit of macrophages to, and poor retention within, lung alveoli. It is therefore interesting to consider that the L-plastin null zebrafish may exhibit a similar response, if tested under appropriate conditions.

Our long-term survey of L-plastin allele carriers suggests that up to one quarter of the null fish produced do not survive. This effect was repeatable, being observed three equivalent knockout lines. The apparent window of susceptibility is before 6–7 weeks of age, which corresponds to the development of a fully adaptive, humoral immune system in this organism. Compared to terrestrial animals, aquatic animals are more likely to encounter infections because they are surrounded inside and outside by a high-density, relatively nutritive medium that both conveys and supports potential pathogens. Although zebrafish lack lungs, the gills perform a similar function and provide a large surface area of exposed tissue. Unsurprisingly, fish macrophages are quite concentrated in this area, where contact with pathogens and pollutants are likely.

The immune processes that might be compromised in *lcp1* mutant macrophages are not known, nor have we documented that poor macrophage motility contributes to premature death in this species. The prospects for discovery in this area are excellent, however, as many zebrafish tissue types can be used to assay leukocyte responses to injury and infection. These include the tail fin, otic vesicle, gut, brain vasculature and swim bladder [63–67]. A distinct advantage of L-plastin null zebrafish for such experiments is that leukocytes within these tissues can be tracked fluorescently *in vivo*, providing additional data on cytoskeletal organization and migration behavior.

### How do L-plastin null organisms regulate the loss of protein and survive?

Although L-plastin is a highly-conserved, highly-expressed, and highly-specific product of normal leukocytes, most null mice and null fish can survive and breed normally. The fact that these animals are not affected indicates that mutant cells can somehow compensate. Compensatory mechanisms could include cytoplasmic recruitment of other actin-bundling proteins, translational activation of existing mRNAs, or genetic upregulation of new ones. Recently, it has been shown that engineered DNA mutations can trigger effective, compensatory gene pathways not activated by transcriptional or translational knockdown approaches [68]. This phenomenon can lead to the appearance of a genetic null animal with no external phenotype. However, the true phenotype is internal, being the novel mechanism that offsets the mutation's effects. In this arena, the null mouse and null fish are now available. Using them, it may be possible to detect which of these mechanisms is activated when the L-plastin is mutated, and if the mechanisms used are similar in both species. L-plastin mutant zebrafish may not show immediate immunological or oncological phenotypes, yet making such knockouts is the first step in the long-term challenge of finding compensatory genes or pathways that regulate the cytoskeleton. The era of single-gene phenotypes is likely over, with the low-hanging fruit already analyzed. Phenotypes that do not depend on a single gene may require double- or triple-knockout animals to test, and making such animals is accelerated with the zebrafish model.

### Future directions for plastin progress in cancer and immunity

We have generated the first L-plastin null zebrafish and independently validated several lines, confirming the absence of protein. These lines are now available to the research community, accelerating the study of actin-binding proteins in health and disease. Two major avenues are apparent, namely cancer and immunity. The aberrant expression of L-plastin in cancers has been appreciated since its discovery 30 years ago in a human, transformed cell line. Subsequent studies have confirmed the importance of L-plastin as a tumor marker, pro-migratory factor, and metastasis driver. Conversely, the complete absence of L-plastin might be protective against the start of tumors or perhaps their spread. To date, we are not aware of any studies of cancer susceptibility in a whole-organism, L-plastin mutant background. These studies are now feasible in zebrafish, which is a common host for endogenous and xenotransplant tumor studies. Because all three of our null alleles can produce viable, homozygous mutant adults, it will be instructive to study the role of L-plastin during different pathological conditions such as inflammation and infection. Given that macrophage motility is the major defect seen in L-plastin null mice, this leukocyte population can now be further tracked in zebrafish, which offers easy access to cell imaging *in vivo*. Overall, our novel mutant lines establish a sound genetic model and an enhanced experimental platform for further studies of L-plastin function in leukocytes and tumors.

## Methods

### Phylogenetic analysis

Our analysis of the plastin proteins used the online software platform Phylogeny.fr [69] and comprised the following steps. A single mRNA reference sequence (zebrafish *lcp1*, see Table 1) was used as a protein-to-nucleotide query in BLASTx, returning a ranked list of matches. Fifteen of the top-ranked proteins were chosen for analysis; for accession numbers, see Fig 2. Amino acid sequences were aligned with MUSCLE v. 3.8.31 [70], configured for highest accuracy. After alignment, regions containing gaps and/or poorly aligned residues were removed with Gblocks v. 0.91b [71]. Using the cleaned blocks as input data, we constructed a maximum-likelihood phylogenetic tree with PhyML v. 3.1 [72]. Within PhyML, we selected the default substitution model, meaning that both the proportion of invariant sites and the gamma parameter used to model substitution-rate heterogeneity were estimated empirically from the input data (proportion = 0.00; gamma parameter = 0.711). Bootstrap replicates (N = 100) were performed to assess the reliability of the branch topology; branches with < 30% bootstrap support were collapsed. The final phylogenetic tree was graphed with TreeDyn v198.3 [73].

### Fish husbandry

All animal work was approved by the Stanley Manne Children's Research Institute Institutional Animal Care and Use Committee or the DePaul University Institutional Animal Care and Use Committee. Adult fish were housed in a 1.4–2.4 L aquaria in a recirculating housing rack at 28°C. The adult diet was a 50:50 mix of finely-ground flake food (TetraMin) and decapsulated brine shrimp eggs (American Brine Shrimp) once or twice a day. Adult fish were stocked at maximum density of 1 fish/200 mL. Fertilized eggs were obtained by natural spawning; healthy clutches were processed for study or propagated for line maintenance. After 5–6 days of development, small fry were transferred to a stand-alone rotifer co-culture system for up to one week [74] before transfer to standard housing. Once in the recirculating system, animals in the study were monitored daily. Dead animals were removed, and sick animals were euthanized if they met certain criteria (*e*.*g*., lethargy, improper buoyancy, emaciation, surface breathing). Euthanasia was by immersion in ice water (2–4°C).

### CRISPR/Cas-9 targeting of lcp-1 exon 2

#### Template assembly and *in vitro* transcription of guide RNA

A gRNA forward primer was designed such that an upstream SP6 polymerase binding site flanked the desired *lcp1* exon 2 target sequence (Table 1, Oligos and Primers). This specific forward was then matched with a generic reverse for high-fidelity PCR amplification (Q5 PCR Kit, New England BioLabs) from a publicly available gRNA template (pT7-gRNA, Addgene #46759) [75]. The resulting 120-bp amplicon was cleaned by phenol-chloroform extraction, and then precipitated by addition of 100% ethanol, 3M sodium acetate, and 1 μL GlycoBlue^TM^. After vigorous centrifugation (20,000 g for 15 min) the purified pellet was air-dried and resuspended in 20 uL of nuclease-free water.

Approximately 4 μg of the purified DNA template was used for a 4x (80 μL) guide RNA *in vitro* transcription reaction according to the manufacturer's protocol (SP6 MAXIscript Kit, Ambion). After 4 hours at 37°C, the template was digested with 4 μL of DNAse for an additional 15 min. The entire reaction was cleaned through a column (Nuc-Away, Ambion), and the flow-through precipitated by addition of 100% ethanol, 3M sodium acetate and 1 μL GlycoBlue^TM^. After one hour at -80°C, the solution was centrifuged for 15 min at 20,000 g to pellet the RNA. After resuspension, concentration and purity were assessed on a Nanodrop spectrophotometer; a separate aliquot was checked for size on a denaturing RNA gel. The remaining guide RNA was frozen in single-use aliquots (5 μL) at -80°C.

#### Egg collection and CRISPR/Cas9 injection

Capped nuclear-localized Cas9 mRNA (nCas9n) was synthesized by *in vitro* transcription from a publicly available plasmid vector (pCS2-nCas9n, Addgene #47929) [75]. Freshly fertilized eggs (1–2 cell stage) from a standard wild type strain (ABTU) were mounted in agar ramps for injection. The injected volume was 1–2 μL of a CRISPR/Cas9 reaction mix (0.3x Danieau buffer with 35–50 ng/μL *lcp1* guide RNA, 100–150 μg/nL nCas9n capped mRNA, and 0.025% phenol red). From each clutch, 5–10 embryos were set aside as uninjected controls. 1–2 days after injection, we collected samples of embryos (at least 8 per clutch) to screen for gene editing. After 5–6 days of development, all normal-appearing larvae were returned to the rearing system.

### Genomic DNA isolation

Genomic DNA was obtained from adult fish by caudal fin clips, and from younger fish by processing whole or bisected embryos. Fish <6 dpf were euthanized on ice before processing. For adult fin clips, fish were sedated using a buffered solution of Tricaine (MS-222, Western Chemical Inc.) in system water (pH 7.5) until the startle response ceased and buoyancy equilibrium was lost. Anesthetized fish were positioned laterally on top of a clean Petri dish and the distal half of the caudal fin was removed with forceps and a clean razor blade. Post-op recovery tanks contained dilute methylene blue as an anti-infection treatment.

The collected tissues were placed in 0.2 mL PCR tubes containing 50 uL of freshly-made DNA extraction buffer (10 mM Tris-HCl, 50 mM KCl, 0.3% Tween-20, 0.3% Triton-X), then heated at 98°C for 10 minutes. 1.0 μL of Proteinase K was added, and the samples were incubated at 55°C until completely dissociated (3–18 hours). Following enzyme inactivation (98°C for 10 minutes), insoluble material was pelleted by brief centrifugation. Unpurified DNA in the aqueous phase was used immediately for PCR genotyping, or stored at -20°C.

### Exon 2 PCR and restriction digest analysis

For PCR genotyping, 2 μL of unpurified genomic DNA was added to 18 μL of PCR reaction mix (BullsEye Taq, MidSci Scientific). Forward and reverse primer sequences are provided in Table 2. After DNA denaturation (92°C for 90 sec), amplification proceeded using touchdown PCR as follows: 10 cycles of (92°C, 20 sec; 68–58°C, 30 sec; 72°C, 40 sec), 33 cycles of (92°C, 20 sec; 58°C, 30 sec; 72°C, 40 sec), and a 72°C hold (2 min).

For enzyme digest, 10 μL of fresh PCR product was combined with 10 U of restriction enzyme (*Bsl*1 or *Mnl*1, New England BioLabs), 1.5 μL of CutSmart buffer, and nuclease-free water (total volume = 15 uL). The reaction was incubated at the optimal restriction temperature for 1–2 hours, and then the products were separated on 3.0–3.5% MetaPhor agarose in electrophoresis buffer (Tris-borate-EDTA) with ethidium bromide. Good separation was achieved using 220V for 25 minutes.

Table 3 gives the expected band sizes produced by each digest. In wild types, the diagnostic PCR product is 175 base pairs long and includes three (3) *Bsl*1 restriction sites (5’–CCNNNNNN|GG–3’). A complete digestion yields fragments of 95, 56, 24, and 1 bp, respectively. In practice, the 1 bp band cannot be seen and the 24 bp band is often faint, so wild type alleles are best recognized as a doublet (95, 56). In CRISPR mosaic animals, some PCR products will have a disrupted *Bsl*1 site due to local indels. Therefore a complete digestion yields a diagnostic larger band, in addition to the smaller bands generated by the normal sequence. Therefore a mosaic animal appears as a triplet (*e*.*g*., 145, 95, 56). All gel images were obtained using the AlphaImager ® gel documentation system, and optimal exposures were saved as TIFF files.

### Subcloning and sequencing

To identify the modified base-pair sequences of the CRISPR/Cas9-generated *lcp1* alleles, undigested exon 2 PCR products from identified mosaic animals were subcloned into a T/A cloning vector (pGEM-T Easy, Promega) and transformed into chemically competent *E*. *coli* cells (TOP-10 One Shot, Invitrogen). Antibiotic-resistant colonies were screened using colony PCR, and positive colonies were expanded into culture. Purified plasmid DNA was sequenced by an outside vendor (Genewiz), and the resulting chromatograms were analyzed using the ApE software (*biologylabs*.*utah*.*edu/ jorgensen/ wayned/ape/)*.

### L-plastin immunohistochemistry

Whole-mount immunohistochemistry followed general procedures for maximizing tissue penetration [76]. Embryos of 5–6 dpf were fixed in cold 4% paraformaldehyde / phosphate-buffered saline (PF-PBS) overnight at 4°C. After rinsing in PBS, the tissues were blocked 2–4 hours with 10% goat serum in antibody solution (1x PBS + 0.5% Triton-X +2% DMSO). The primary antibody, a rabbit polyclonal against zebrafish L-plastin, was the kind gift of Dr. Michael Redd. This reagent was diluted 1:20,000–1:50,000 in antibody solution and applied to the tissues for 1–3 days at 4°C. After 5–6 rinses in PBS, the tissue was incubated in a fresh aliquot of antibody solution containing a red fluorescent secondary (1:200, goat anti-rabbit Cy3, Jackson ImmunoResearch) and a nuclear counterstain (1:1000, 5 mg/mL DAPI in DMSO). After 1–3 days at 4°C in the dark, the embryos were rinsed 4–5 times in PBS, cleared in 50% glycerol and mounted in glycerol-compatible anti-fade medium (CFM-Mount, Electron Microscopy Sciences). Confocal stacks were obtained with a Zeiss LSM 800 and processed using Zeiss Efficient Navigation (‘ZEN’) imaging software.

### Detection of neutrophils and macrophages

For neutrophil detection we used the histochemical substrate Sudan Black [see also 44, 77]. 20 mg of Sudan Black stain (#21610, Electron Microscopy Sciences) was combined with 100 mL of 70% ethanol and 200 uL of buffered phenol-chloroform-isoamyl alcohol. After stirring for 2 hours, the mixture was filtered and stored at room temperature for up to four months. Embryos were treated with 1-phenyl 2-thiourea (PTU) in egg water to prevent pigment development [78]. At 2 days post-fertilization, treated embryos were dechorionated manually and fixed in 4% PF-PBS at room temperature for 2 hours, followed by rinsing in several changes of PBS-T. For staining, approximately 30–50 embryos were placed in a 1.5 mL tube. The supernatant was removed, ~1 mL of Sudan Black stain solution was added, and the mixture was agitated on a shaker platform for 30 minutes at room temperature. After several washes in 70% ethanol, the embryos were rehydrated in PBS-T and cleared in 50% glycerol.

For macrophage detection we crossed *lcp1* homozygous mutants (FX -/-) for two generations into the Tg(*mpeg1*.*1*:Dendra2) transgenic line [79]. This produced 50:50 clutches of heterozygous:null embryos, half of which had green macrophages. Eight 2-dpf embryos were collected at random, euthanized, and then bisected for microscopy and genotyping as described above. Confocal stacks were obtained on a Leica SP5 microscope and processed with FluoRender software [80].

### Protein extraction and quantification

Total protein was obtained from pooled zebrafish embryos (2 dpf) and individual adult fin clips. Prior to protein extraction, embryo chorions and yolks were removed. For chorion removal, we added 1 mL of 20 mg/mL pronase per Petri plate of egg water [78]. After 3 to 5 minutes of digestion at room temperature, the solution was pipetted vigorously with a pulled glass pipette to rupture the chorions and release the embryos. Excess enzyme solution was then decanted and replaced with clean egg water 2 or 3 times. For de-yolking, undamaged embryos were transferred to a small dish containing buffered Tricaine in egg water until movement ceased. We then manually ruptured each yolk sac with microtools, working in batches of 30–40 embryos at a time. After dissection, each batch of embryos was transferred to a 1.5 mL microfuge tube on ice and resuspended in 500 uL cold 1x PBS. Vigorous aspiration through a pulled glass pipette released most of the remaining yolk, forming a cloudy supernatant. This wash was repeated at least twice, until the solution became clear. After brief pelleting by centrifugation, the embryos were kept on ice until homogenization, as described below.

For adult fin clips, fish were first anesthetized in buffered Tricaine mixed with system water and then a small piece of the caudal fin was removed. The fin was then transferred to cold PBS in a 1.5 mL microfuge tube, minced using scissors, and spun down to pellet the fragments.

Tissue pellets were resuspended in 100–200 uL of protein extraction buffer (Cytobuster™, Novagen) freshly mixed with a 1:100 dilution of protease inhibitor (Cocktail Set 1, Calbiochem). Tissue was then homogenized for 10–15 seconds on ice using a motorized mini-pestle and disposable tissue grinders (Pellet Pestle™, Kontes). Post-grinding, tubes were rocked for 5 minutes at room temperature, followed by a clarifying spin (5 min >15,000 g at 4°C). Cleared supernatants were stored in aliquots at -80°C.

### Protein quantification

Cleared protein supernatants were serially diluted in Cytobuster™ and then quantified using a commercial assay kit (Micro BCA™, ThermoScientific). Six protein standards (bovine serum albumin, 5–100 mg/mL) were processed in parallel with each set of samples, providing a calibration. After a 2-hour reaction period, the 562-nm absorbances of the standards and samples were measured on a Nanodrop 2000c (ThermoScientific). Sample absorbances above or below the linear range of the standards were omitted from any calculations.

### SDS-PAGE and Western blotting

Proteins were separated using standard denaturing PAGE. Using the procedure below, we found that 3–6 μg of total protein per lane was adequate for chemiluminescent detection. Briefly, protein lysates were thawed, diluted in Cytobuster™, and mixed 1:1 with 2x Laemmli sample buffer (BioRad) + 5% β-mercaptoethanol (final concentration = 1x Laemmli, 2.5% βME). After 5 minutes at 95°C in a thermal cycler, the denatured samples were loaded into a pre-cast polyacrylamide gel (10% Mini Protean TGX, BioRad). For band sizing, we loaded in a separate lane 10 uL of a pre-mixed, wide-range protein marker (Precision Plus WesternC, BioRad). Separation was at 200V for 50–60 min in a Tris-glycine-SDS buffer (pH = 8.3). During longer runs, the three smallest, blue-colored standards (10, 15 and 20 kDa) would migrate out of the gel and into the buffer. This longer migration produced better separation between L-plastin (65 kDa), actin (43 kDa), and several non-specific bands in the samples.

Proteins were transferred to PVDF membrane (0.2 μm Immun-Blot, BioRad) that had been pre-soaked in methanol and equilibrated in transfer buffer (25 mM Tris, 192 mM glycine, 20% methanol, 0.025% SDS, pH = 8.3). Transfer was performed in an ice-cooled Criterion blotter apparatus at maximum current (400mA) for 75 minutes. After transfer was complete, the blot was rinsed in distilled water and air-dried for up to one hour. If not used immediately, the blots were stored in sealed bags at 4°C. Prior to detection, dry blots were dipped briefly in methanol and equilibrated in TBS-T (= 1x TBS, 0.01% Tween-20). Blocking was in TBS-T + 3% BSA for 1–2 hours. Primary antibodies were diluted in TBS-T at the following concentrations: rabbit polyclonal anti-zebrafish L-plastin 1:50,000 (kind gift of M. Redd); mouse monoclonal anti-actin 1:400 (JLA20, Developmental Studies Hybridoma Bank).

After overnight incubation at 4°C, the blots were then rinsed with TBS-T three times for 10–15 min each. Secondary antibodies were diluted in TBS-T at the following concentrations: Immun-Star^TM^ goat anti-rabbit HRP 1:20,000 (BioRad); goat anti-mouse HRP 1:10,000 (Jackson ImmunoResearch). For chemiluminescent marker detection, we added an additional HRP conjugate (1:10,000 Precision Protein StrepTactin-HRP, Bio Rad) to each secondary antibody solution. After overnight incubation at 4°C, blots were rinsed with TBS-T twice for 5–10 minutes each, then once with TBS lacking detergent for 5 minutes. A chemiluminescent substrate solution was prepared (Clarity Western ECL Substrate, BioRad) and applied to the blot surface for 5 minutes. Exposures of 2–10 minutes were captured on a dedicated imager (Li-Cor Odyssey FC) and analyzed using the manufacturer’s software (Image Studio Lite Version 5.2). Post-exposure, transfer efficiency and equal loading were assessed on both gels and blots using standard Coomassie Blue techniques.

### Statistical analysis

Observed and expected genotype frequencies were compared using chi-squared analysis and the 95% confidence interval on a proportion. Statistical analysis and data graphing were performed in Prism 6.0 (GraphPad Software, Inc.). Differences were considered significant at a probability value of p <0.05.
